# “Poison to the Economy”: (Un-)Taxing the Wealthy in the German Federal Parliament from 1996 to 2016

**DOI:** 10.1007/s11211-021-00383-y

**Published:** 2022-01-29

**Authors:** Till Hilmar, Patrick Sachweh

**Affiliations:** SOCIUM–Research Center on Inequality and Social Policy, University of Bremen, Mary-Somerville-Str. 9, 28359 Bremen, Germany

**Keywords:** Discourse, Inequality, Taxation, Social justice, Wealth

## Abstract

The concentration of wealth is a key component of the rise in economic inequality at the beginning of the twenty-first century. While the abolition of taxes on private wealth during the 1990s and 2000s is recognized as an important institutional driver behind this development, comparatively little is known about the justification of tax cuts for the wealthy in advanced democracies. This paper investigates how the abolishment of the personal net wealth tax in Germany, a country with high levels of wealth inequality, has been debated and justified in parliament over a period of 20 years. Using a mixed methods approach that combines computational social science methods and a qualitative analysis, we examine how Germany’s two major parties, the Christian Democrats (CDU) and the Social Democrats (SPD), have variously construed the meaning and purpose of the wealth tax and justified their support for or opposition to it. While the Social Democrats debate the wealth tax primarily from a social justice perspective, the Christian Democrats rely on an efficiency frame that invokes biological metaphors, enabling them to narrate the wealth tax as a threat to the social body. Paradoxically, then, by arguing that the tax is “poison to the economy”, conservative discourse succeeds in linking opposition to the wealth tax to a principle of social unity. On these grounds, we suggest that future research should scrutinize how the interrelation between political discourse and institutional architectures has facilitated the rise of wealth inequality in recent decades.

## Introduction

The increasingly unequal distribution and concentration of wealth is a major component of the rise in economic inequality across many OECD countries at the beginning of the twenty-first century (Alvaredo et al., 2017; Balestran & Tonkin, 2018; Piketty, 2014). Since the 1990s, the wealth share held by the richest 10 per cent of households has been increasing in Europe and the USA (Piketty & Saez, 2014: 839). In 2015, the wealthiest 10 per cent of households in the OECD on average held 52 per cent of total net wealth, while the least wealthy 60 per cent held just about 12 per cent (Balestran & Tonkin, 2018).

Besides rising top-end income inequality, the abolition of taxes on private wealth in the 1990s and 2000s has contributed significantly to this growing concentration of wealth (Hacker & Pierson, 2010; Piketty, 2014; Saez & Zucman, 2019; Swank, 2016). While the institutional and political drivers behind tax cuts for the wealthy are increasingly well understood (Glennerster, 2011; Hacker & Pierson, 2010; Saez & Zucman, 2019; Scheve & Stasavage, 2016), comparatively less is known about how these developments have been justified vis-à-vis the non-wealthy majority of the population in rich democracies (but see Scheve & Stasavage, 2016). After all, under democratic conditions, the poor and the middle classes could use their numerical strength to impose egalitarian policies, such as wealth taxation, on the rich (Acemoglu & Robinson, 2008; Meltzer & Richard, 1981). Moreover, the personal net wealth tax is of great symbolic relevance as it reflects the “commitment of the state to redress some of the more egregious inequalities generated by the market economy” (Bird 1989: 441, cited in Banting, 1991: 352). Therefore, understanding how tax cuts favouring the well-to-do have been legitimized in an era of rising inequality is not only politically important but also of great theoretical significance.

Against this backdrop, this paper asks how the abolishment of the personal net wealth tax (in the following, abbreviated as “wealth tax”) in Germany has been debated and justified in the parliamentary debate. Germany represents a particularly interesting case to study political debates over wealth taxation for several reasons. First, wealth inequality in Germany is higher than in other European countries. According to latest estimates, the wealthiest 10 per cent of households own more than two-thirds of total net wealth (Schröder et al., 2020). At the same time, issues of wealth and its concentration are rarely a matter of public debate, as the wealthy remain secluded and are hardly ever publicly visible (see Theine & Grisold, 2020). Therefore, analysing parliamentary debates over wealth taxation can enhance our understanding of how the sources of economic privilege are selectively revealed as well as discursively concealed. Second, the wealth tax has not been levied since 1997 due to a ruling by Germany’s highest court (the Federal Constitutional Court) on the unequal treatment of real estate and financial wealth and has not been reinstated since. This abandonment occurred during a critical period of redistribution to the top in Germany, when capital taxes were slashed while an ever-increasing share of the national income went to the top 10 per cent whereas lower- and middle-class wages stagnated (Corneo et al., 2014; Groh-Samberg, 2016). Surprisingly, the coalition government of the Social Democrats and the Green Party that existed from 1998 to 2005 did not follow up on its initial plans of implementing a constitutionally compliant reform of the wealth tax.

Our investigation contributes to an emerging literature that analyses how issues of wealth taxation are publicly debated. While prior studies have mostly focused on public discourse in the media (Leipold, 2019; Lichtenstein et al., 2016; Rieder & Theine, 2019), our study is the first to analyse the parliamentary debate over time. In modern democratic polities, the parliament is an important site of communication, deliberation, and coordination, both between different political actors as well as vis-a-vis the larger public (van Dijk, 1997). Hence, investigating how the taxation of wealth is debated in parliament can yield important insights into the arguments and justifications advanced by political actors to defend their support for or opposition to it.

To examine how Germany’s two major parties, the Christian Democrats (CDU) and the Social Democrats (SPD), have variously construed the meaning and the purpose of the wealth tax, we use a mixed methods approach that combines an exploration of parliamentary debates from 1996 to 2016 based on computational social science methods (Blätte & Blessing, 2018; Manning et al., 2008) and a qualitative analysis. We find that two competing frames (DiMaggio et al., 2013)—an economic efficiency frame and a social justice frame—variously imbue the wealth tax with meaning. While prior research finds that in public discourse on the taxation of wealth, purported negative economic implications are often invoked to underline opposition to this measure (Leipold, 2019; Lichtenstein et al., 2016; Rieder & Theine, 2019), we need more systematic evidence for how arguments that are crafted in the political sphere might resonate with a broader public. Our findings suggest that evocative language and political symbolism may play a crucial role in this regard: in the parliamentary debates we analyse, Conservatives’ use of the efficiency frame often rests on biological metaphors. They consistently narrate the wealth tax as a threat to the social body, arguing that society has to be protected from its detrimental, sickening effects. The tax, they posit, is “poison” to the economy. Conservative discourse thus successfully links opposition to the wealth tax to a principle of social unity. Social Democrats, in turn, only selectively activate the social justice frame, and even in doing so they hardly achieve to account for why taxing wealth would be in the interest of society as a whole.

The paper is structured as follows. The “Conceptual and theoretical background” section outlines the conceptual and theoretical framework of the paper by describing the institutional development and the partisan politics of wealth taxation of wealth in Germany. Thereafter, the “Data and Method” section describes the data and our mixed methods approach to study the evolution of parliamentary debates over the wealth tax in Germany from 1996 to 2016. The “Findings” section distinguishes three phases of the debate on the wealth tax in the German federal parliament and analyses the frames activated in them, and the concluding “Discussion and Conclusion” section discusses the implications of our findings for contextualizing the globally resurging debate on taxing wealth today.

## Conceptual and Theoretical Background

### Wealth Taxation in Germany

Before discussing the positions of the two major political parties on the taxation of wealth, we briefly review the role and historical development of the wealth tax in the context of the German conservative welfare state. The taxation of wealth in Germany dates back to the late nineteenth century, when the *Verein für Socialpolitik* laid out the need for a tax on wealth,^1^ arguing that taxes on income are insufficient as they do not reflect the actual material possessions (and the benefits accrued through them) of individuals (cf. Wieland, 2003).^2^ Prussia was the first German federal state to levy a wealth tax on these grounds. In order to cope with the fiscal burden of World War I, Matthias Erzberger, the Social Democratic minister of finance in the Weimer Republic, implemented a series of tax reforms in 1919 and 1920 that subsequently extended the wealth tax across all federal states (Bach & Buggeln, 2020).

After the Second World War, the CDU-led government under chancellor Konrad Adenauer continued to levy an annual personal net wealth tax, set at a rate of 0.75 per cent (Wieland, 2003: 13).^3^ It was accompanied by a law on the “Equalization of war burden” (“Lastenausgleichsgesetz”), which also included a one-time levy on wealth of 50 per cent (to be paid off over 30 years) as a redistributive mechanism intended to compensate for war damage to property (cf. Scheve & Stasavage, 2016: 189). The rate of the annual net wealth tax was changed several times under Social Democratic (0.7% in 1977) as well as Christian Democratic governments (0.6.% in 1983) and was set at a rate of 1 per cent for natural persons when it was last levied in 1996 (Scholz & Truger, 2013: 35ff.). In 1995, the Federal Constitutional Court declared the unequal treatment of real estate and financial wealth unconstitutional, and as a consequence the wealth tax has not been levied since 1997.

Within the institutional architecture of Germany’s conservative welfare regime, the extent of redistribution between rich and poor is limited (Bradley et al., 2003; Esping-Andersen, 1990). Whereas the social insurance schemes primarily achieve a horizontal redistribution of income flows between life-phases, the effects of the German tax system on redistribution between different income groups are moderate. Extant wealth-related taxes, such as property or inheritance taxes, target the (upper) middle class rather than the super-rich (Bach, 2016: 74), and taxes on capital—for example on business profits or capital gains—are relatively modest (Prasad, 2006: 167). Nevertheless, some estimates suggest that a reintroduction of an annual personal wealth tax could yield a potential revenue of around € 10 to 20 billion Euros—even with generous tax allowances—and lead to a slight reduction in income inequality as a result of the taxation of income generated from wealth (e.g. interest, dividends) (Bach & Thiemann, 2016: 83). While economists often associate wealth taxation with negative economic consequences (Fuest et al., 2017), recent research establishes that tax cuts for the rich increase inequality while having no positive effects on economic outcomes, such as growth or employment (Hope & Limberg, 2020).

### Political Discourse and Partisan Positioning Towards Wealth Taxation

Why do political debates around the taxation of wealth matter? Several strands of the literature emphasize the role of ideas and culture for welfare state development and reform (Pfau-Effinger, 2005; Sachweh, 2011; Schmidt, 2008; Steensland, 2006). Most pointedly, the impact of ideas on institutional change and stability is highlighted by scholars who make use of the discursive institutionalism approach (Schmidt, 2002, 2008). While discursive institutionalists do not discard economic conditions, power resources, or institutional arrangements as explanatory factors, they maintain that the success of any given policy proposal depends on whether it is rooted in substantive ideas that resonate with an audience. Political actors must accept and communicate the legitimacy of a policy proposal among themselves as well as towards the public (Schmidt, 2008). This involves a discursive exchange about the cognitive and normative content of the paradigmatic ideas and worldviews which, in the form of unarticulated, taken-for-granted “background knowledge” (Schmidt, 2008: 308), underpin specific policies. We build on this insight in our analysis of parliamentary debates by reconstructing the frames and modes of causal reasoning which underlie political parties’ support for or opposition to the wealth tax.

We analyse political discourse as a form of public discourse (van Dijk, 1997). In political discourse, actors are embedded in particular institutional contexts, and their respective institutional role also defines their agency. Speaking in parliament, political actors aim at convincing the public of their policy positions and to form majorities on the floor; at the same time, they are accountable to (and thus subject to pressure from) their political fractions and groups. Hence, they are simultaneously involved both in a “coordinative discourse” within the policy sphere and a “communicative discourse” directed at the broader public (Schmidt, 2008: 310). While they do not speak as private citizens, they nevertheless draw on a shared symbolic and affective language that reaches beyond the sphere of “the political”, invoking citizens’ everyday life and popular forms of reasoning. Parliamentary speech is therefore also a form of justifying policy positions. Legislative accountability comes with the fact that reasons for supporting or opposing particular policy proposals are provided, made transparent, and recorded for the public to see.

Within parliamentary speech, we trace dominant frames of meaning. In Goffman’s (1974) classic definition, frames help actors to make sense of a situation by invoking a larger structure of meaning. DiMaggio et al., (2013: 539) define a frame as a “set of discursive cues (words, images, narrative) that suggests a particular interpretation of a person, event, organization, practice, condition, or situation.”^4^ On these grounds, dominant frames can be understood as organizing principles for a set of discursive cues that repeatedly occur together with the term “wealth tax”. We assume, furthermore, that frames advanced around this topic target the very rationale for having (or not having) such a tax (making opposition to or support for the tax seem like the most natural and reasonable choice) and thus go far beyond the mere technical issues commonly associated with fiscal policy.

What are the major ideological traditions and cultural repertoires different political parties draw upon in framing their position towards the taxation of wealth? And in what ways do these reflect their electoral base and the social groups they represent? Conservative parties typically combine an ideological preference for limiting the reach of government with economically liberal positions (Peters, 1991; Prasad, 2006). This implies that state intervention in the economy should be minimal and foster business-friendly conditions. With regard to issues of taxation, conservative parties can therefore be expected to argue for lower taxes on income as well as private and corporate wealth holdings, while relying more strongly on regressive forms of taxation (e.g. sales taxes) (Peters, 1991; Saez & Zucman, 2019). Electorally, they—together with liberal parties—represent politically powerful segments of the population, including not only wealthy individuals but also the corporate and financial sector, small business owners, family businesses, farmers, and (upper) middle-class taxpayers anxious about an extension of wealth taxes to their fortunes (Banting, 1991: 354). As these groups are well organized in strong lobby organizations, the taxation of wealth is politically costly for conservative parties. In Germany, the Christian Democrats (CDU) are the major conservative political force, and together with the economically liberal Free Democrats (FDP), they have formed the ruling coalition government from 1982 until 1998 and from 2009 until 2013. For most of the period from 2005 until 2020, the Christian Democrats have been in government in a so-called “Grand Coalition” with the Social Democratic Party (SPD). We assume that the Christian Democrats oppose attempts to retain or reintroduce the personal net wealth tax and voice their opposition against it primarily on the grounds of economic efficiency.

By contrast, Social Democratic parties have traditionally been in favour of government intervention into the economy and endorsed an egalitarian ideology emphasizing redistribution between rich and poor (Peters, 1991). “Parties of the left attempt to focus attention on inequality in the distribution of wealth, income and power, and to convince voters of the importance of redistribution through a variety of instruments, including the tax system” (Banting, 1991: 355). The “power resources” approach (Esping-Andersen & Korpi, 1984; Korpi, 1983) therefore considers the strength of organized labour—as indicated by the power of Social Democratic parties or trade unions—as a major determinant of redistributive public policies (Bradley et al., 2003; Brady, 2006; Castles & Obinger, 2007; Esping-Andersen, 1990). Thus, parties on the political left would be expected to advocate for the levying of a personal net wealth tax based on arguments about fairness and social justice.

However, during the 1990s and 2000s, a transition in Social Democratic parties’ economic policy experts has engendered a shift from a “Keynesian ethic” to a “neoliberal ethic” (Mudge, 2018). In line with “third-way” approaches to social and economic policy making (Giddens, 1998), the goal of promoting and preserving the functioning of markets, rather than a Keynesian steering of the economy, has increasingly gained traction among Social Democratic parties across Western Europe (Mudge, 2018; Rademacher, 2017). Furthermore, “social investment” approaches that emphasize the role of social policy for facilitating and enabling employment have gained prominence as Social Democrats have attempted to attract a broader constituency of middle-class voters (Gingrich & Häusermann, 2015).

Still, we assume that the major German party on the centre-left would favour a tool of wealth taxation on the grounds of a redistributive logic. Within the German political system, the Social Democratic Party (SPD) has for a long time represented the dominant force on the political left, but it is complemented by the Green Party since the 1980s, as well as by the Left Party (“Die Linke”) since the mid-2000s.^5^ Traditionally, the German Social Democrats have been major supporters of welfare state expansion and egalitarian policies aimed at redistribution. Yet, under the Red-Green government led by chancellor Gerhard Schröder from 1998 until 2005, the Social Democrats have introduced a series of “third way”-inspired welfare reforms that were in line with the neoliberal spirit of that time (Mudge, 2018) and that initiated significant path-departures within the institutional architecture of Germany’s conservative-corporatist welfare state (Streeck, 2009).^6^

### Previous Research on Debates Around Wealth Taxation

In previous research, a small but growing strand of the literature explores public debates about wealth taxation in German media outlets. One key finding here is that arguments that emphasize potentially negative economic consequences of taxing wealth—for growth, employment, etc.—are a prominent trope. For instance, Lichtenstein et al. (2016) study media representations in 2013, an election year, and find that worries about negative economic consequences of the wealth tax are a dominant theme across different types of media outlets, over and above favourable arguments around its redistributive effects, considerations about how the revenue could be used, and legal issues concerning its feasibility as well as its implementation. Rieder and Theine (2019) investigate how the policy proposals put forward by French economist Piketty are portrayed in the media in Germany and four additional European countries in 2014 and 2015. Focusing on counter-arguments to Piketty’s suggestions, the authors find a set of highly symbolically charged arguments that focus on negative economic consequences of taxing wealth as well as on discursive strategies such as portraying “the rich and entrepreneurs as victims lacking agency in the face of an aggressive, overpowering and abusive state” (ibid. 260). Similarly, Leipold (2016) analyses the discourse network on the wealth tax in two major German newspapers from 1995 to 2015. He documents how, throughout this period, a hegemonic discourse coalition pressed for keeping taxes on wealth and capital low by citing international tax competition and the need to consolidate national budgets (Leipold 2016: 92). Overall, these studies demonstrate that negative depictions of measures that aim to alleviate wealth inequality are relatively widespread in the media.

Going beyond media debates, Rademacher (2017) studies the justification of income tax cuts for high incomes in parliamentary discourse (in addition to presidential addresses and debates in political committees). She finds that growth narratives which highlight positive “trickle-down” effects of cutting taxes for top income earners are successful in convincing other political actors only when they are moralized by arguing that this generates a universal common good (Rademacher, 2017: 31, 2021). In the following, we build on these recent forays into discursive constructions and legitimations of economic inequality (Smith Ochoa & Hugendubel, 2019; Grisold and Preston 2020). Specifically, we contribute a longitudinal perspective as we assume that the ideational resources that actors draw upon to justify or dismiss particular redistributive policies can be best understood by investigating shifting justifications and contestations of inequality over time. This allows us to consider recurring motifs in the way actors frame the taxation of wealth.

## Data and Method

We analyse speeches on the wealth tax held in the German Federal Parliament between February 1996 and December 2016. We draw on the GermaParl corpus (Blätte & Blessing, 2018), an annotated collection of all parliamentary speeches delivered in the German Bundestag in this timeframe.^7^

To approach this vast trove of data with a specific focus, we examine how the two largest parliamentary groups, the Christian Conservatives (CDU/CSU) and the Social Democrats (SPD), each discuss the wealth tax in parliament.^8^ Specifically, we ask: How do these actors talk about the wealth tax differently; what makes their respective vocabulary distinct? The assumption is that unique vocabulary (unique with respect to semantic associations, but appearing regularly) is a pattern of distinct, meaningful speech. It is possible to trace dominant frames that imbue a given word (in German, “wealth tax” is a compound) with meaning. This can be operationalized by examining its semantic neighbourhood—what varying company this word keeps over time.^9^

The advantage of analysing parliamentary speeches on this topic is that they have greater public visibility than any other legislative activity. What is more, even if the wealth tax was, prior to its abolishment, levied by the German federal states and not by Berlin, the issue has since still avidly been contested in the national parliament. Yet, there are also limitations that come with the use of this data. The main limitation is that we cannot systematically consider the discursive role of extra-parliamentary actors, such as think tanks, trade, and labour organizations, in supplying resources and discursive strategies and possibly influencing the legislative process. We also do not cover intra-party conflicts around this issue, even if party elites at the federal level (especially from the wealthy German south) have for a long time played a key role in these debates.

We draw on a computational social science framework—specifically, a text-as-data approach^10^—that allows to reconstruct the semantic associations of the term “wealth tax” relative to the entire corpus (all parliamentary speeches given by parliamentary groups on all topics between 1996 and 2016) and to systematically compare speeches on this topic delivered by different actors. Overall, we follow an inductive approach: Through the statistical analysis of corpus linguistics (see below), we discover patterns of associations between the term “wealth tax” and certain words. We use these associations to identify particularly distinct segments of speech, which we then analyse and compare qualitatively. (We elaborate on the qualitative analysis further below.) Considering context and change over time, it becomes possible to reconstruct frames as recurring patterns of meaning around this term.

## Findings

### Mapping Frequencies

In a first step, we map the term and its usage in a semantic field and over time. The GermaParl package allows to remove interjections to avoid bias (Blätte & Blessing, 2018); all frequencies reported in the following exclude interjections. In total, the term “wealth tax” (“Vermögensteuer”) appears 1832 times during the period from February 1996 to December 2016.^11^

**Fig. 1:**
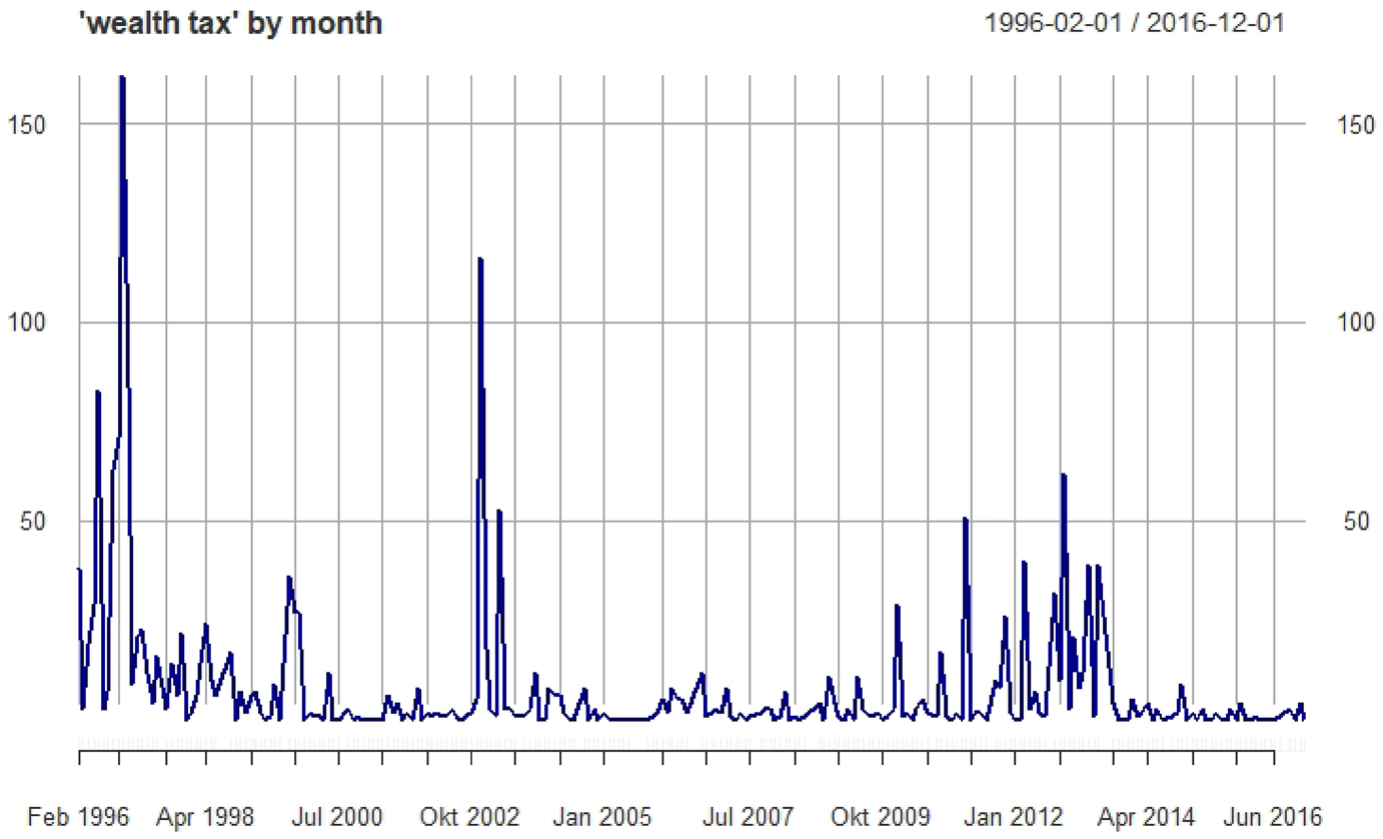
“wealth tax” by month

Figure 1 shows the distribution of this term over the entire corpus (of speeches by all parliamentary groups, “Fraktionen” of the German Bundestag).^12^ We use this information to break down our analysis into three distinct periods: Phase 1 from 1996 to 2001; Phase 2 from 2002 to 2007, and Phase 3 from 2008 to 2016. Periods one and two are marked by a spike in the debate, the final period has a more balanced distribution. In election years (1998, 2002, 2005, 2009, and 2013) the number of parliamentary speeches is much lower than in regular years; consequently, the corpus in these years is smaller (see Blätte & Blessing, 2018).^13^

We focus on the two largest parliamentary groups, the Christian Conservative CDU and the Social Democratic SPD in the following. From Phase 1 to Phase 2, there is a marked decrease in the use of this term; this trend is, however, much more pronounced among Social Democrats. There is a recovery of the issue in the third phase, arguably stronger for the SPD relative to its decline in the second phase^14^ (Table 1).

**Table 1:** Number of times the two major parliamentary groups use the term “wealth tax” in speeches in the three phases

	CDU/CSU	SPD
Phase 1:1996–2001	278	267
Phase 2: 2002–2007	159	60
Phase 3:2008 to 2016	176	106
Total (all parliamentary groups)	613 (1832)	433 (1832)

These trends offer some preliminary insights into the political dynamics around the debate on the wealth tax. The tax was abolished in 1996, after a fierce debate among these two leading political forces. Further attempts to reintroduce it have failed, we can accordingly see the debate evaporate and never truly return to its initial momentum during the timeframe analysed here. These frequencies, however, cannot provide any information about how the nature of the debate has evolved over time, or what particular meanings actors imbue the concept with. To trace this question is our goal in the following section. We ask: How do these actors talk about the wealth tax—what makes the language they use to argue in favour or against this tax *distinct* in each case?

We create a topically defined sub-corpus for each of the two actors in each phase, generating six corpora in total. To reduce the amount of data for the qualitative analysis in transparent way and to capture the distinct language used by the two parties, we apply the following criteria for each of them: In a first step, we only include all dates when the term “wealth tax” was mentioned at least four times; in a second step, we include only the speakers who mentioned the term at least three times on these dates (Table 2). These corpora are then systematically compared using a standard measure in corpus linguistics, the Chi-Square Test (cf. Blätte & Wüst, 2017: 213–214; Manning et al., 2008: 169–171).^15^ Comparing a corpus of interest (COI) to a reference corpus (REF), the test determines whether a word appears more frequently than simply by random chance—whether a word is truly distinct for the corpus of interest in contrast to the reference corpus. The Chi-Square test does not provide mere frequencies, but instead identifies words that are significant in a particular corpus even if their frequency is comparably low.

**Table 2:** Size of the six topically defined corpora

		CDU/CSU	SPD
Corpus Phase 1	Number of tokens	10,951	128,186
	Number of dates	20	21
	Number of speakers	17	17
Corpus Phase 2	Number of tokens	57,306	10,908
	Number of dates	8	5
	Number of speakers	13	6
Corpus Phase 3	Number of tokens	107,660	24,689
	Number of dates	15	7
	Number of speakers	16	10

For each of the corpora gained in this way, the highest scoring words are identified.^16^ We assume that these words have a critical function in the neighbourhood of the term “wealth tax”. We examine them in context: For each corpus, we consider the segments of the plenary speech in which the highest scoring words appear next to the term “wealth tax” (80 segments in total).^17^ We code these segments qualitatively and compare different segments around single words.^18^ Initially, we looked for arguments for or against the wealth tax in the segments. After a first round of coding, it became evident that oftentimes, Members of Parliament do not reason about taxation in a comprehensive, coherent way. Instead, they tend to invoke a particularly important social process or datum—like economic growth, or the status of families in society—and then suggest that it will either be positively or negatively affected by a wealth tax. In other words, they make implicit assumptions about causality in their statements, conjuring narrative elements of evaluation and causal attribution (Somers, 1994). Therefore, we coded the segments with a focus on how actors depict what the tax supposedly *causes*, or what the purported *impetus* for having such a tax might be.^19^ We also document formal features of language that are an integral part of reasoning through narrative, such as metaphors. Comparing segments over time provides a window into the “background knowledge” (Schmidt, 2008: 308) that political actors mobilize when making certain claims. We derive frames of meaning (De Vreese & Kandyla, 2009; DiMaggio et al., 2013) around the taxation of wealth from the comparison over time. The quotes presented in the following are taken from these segments and were translated by the authors.

Juxtaposing the language about the wealth tax *that is most distinct to two major parties* constitutes a methodological choice that also affects the qualitative analysis. The segments are exclusively generated from our two main parties’ corpora of speech. We map the central political tension between these two actors; hence, we necessarily also deemphasize external dynamics in this debate.

### Phase 1, 1996–2001: A time of Contestation

The initial phase is a time of heated debate—first, about the question of whether the wealth tax should be abolished or not, and second, after 1997, about its possible reintroduction. The constitutional court ruling of 1996, to be sure, did not mandate the tax to be abolished, it only declared that it was unconstitutional in its present form. Overall, Phase 1 is marked by a consistent set of arguments against keeping the tax in place from the Christian Democratic side—and a somewhat fragmented plurality of arguments in favour of the tax advanced by Social Democrats.

**Fig. 2:**
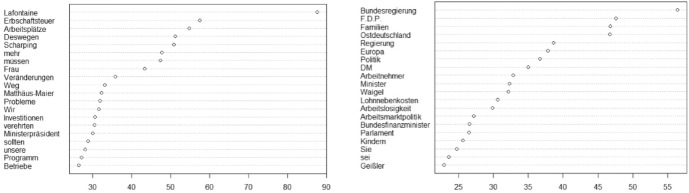
Chi-square results phase 1 (left: CDU/CSU, right: SPD)

Conservatives, who are in power until 1998, repeatedly address SPD party leader at the time Oskar Lafontaine (see Fig. 2), who voiced his opposition to abolishing the tax, to remind him and his party of the harsh reality of globalization. They offer a narrative about how globalized, competitive markets, and specifically, the mechanism of capital flight within them, necessitate national governments to reduce the tax burden:

The following argument must be considered, above all, Mr. prime minister Lafontaine: We talk about financial markets, the flight of capital, the globalization of markets; about the fact that we are in competition with every other site of production in the world, that distances have become less significant, and that capital will look for investment opportunities wherever they are most rewarding. (Wolfgang Schäuble, 1996–02-08).

In this trope, the claim that taxes obstruct competition and thus hurt economic growth is presented as a law-like mechanism. Conservatives suggest that Social Democrats—Oskar Lafontaine is repeatedly referenced as a vocative—still have to grasp this fundamental insight into how markets work.^20^

In these early speeches, Conservatives achieve to link the competition trope to a much broader concern: They claim that German jobs depend on it. The term “jobs” comes third in the list of distinct vocabulary used by CDU/CSU in this phase (see Fig. 2).^21^ Since the wealth tax is a tax on capital, not on labour, this linkage is not intuitive at all. Yet it reveals that, when talking about the need to abolish the wealth tax, Conservatives really offer a much larger narrative about society. In it, they specify a mechanism of capital flow as the lifeblood of the economy—capital is what guarantees investment by as well as in firms, and thus ultimately, in labour. If investments are endangered and capital flows are obstructed, jobs are in danger, as conservative MP Gerda Hasselfeldt asserts in November 1996:

The wealth tax has to be paid from income that was already taxed. In years of loss, it even has to be paid from a company’s capital base. This is an enormous threat to existing jobs and an enormous threat in the area of creating new jobs. It especially affects firms in the start-up phase, particularly in Eastern Germany. Capital will go wherever it pays to go. Along with capital, investments will go wherever it pays to go. Along with investments go jobs. The global competition taking place not just in our country but around the world, does not tolerate redistributive political ideologies, like the ones happening in Germany from time to time. (Gerda Hasselfeldt, 1996–11-07).

The theme emerges as a general pattern: In rejecting the wealth tax, CDU/CSU invokes a language of fragility, protection, and safety, arguing that there is a need to protect the flow of capital in order to salvage jobs from disappearing. Firms—especially young firms that need fresh capital—are vulnerable. Since the wealth tax targets a firm’s capital stock independent of profits in a given year, Conservatives argue, it may “devour” or “annihilate” a firm’s assets (“substanzverzehrend”, “substanzvernichtend”, Peter Rauen, 1996–06-14) in times of low yield and thus effectively runs the danger of inflicting irreparable damage to these firms. Distinct speech regularly references mid-tier and family businesses (the German “Mittelstand”) in conjunction with this set of arguments.^22^ Conservatives warn that, if the state fails to protect them, it ultimately fails to act in the interest of both employers and employees. Effectively, then, they argue that ending the wealth tax is in the interest of the economy as a whole, not of particular groups in society.

Another recurring argument advanced in favour of abolishing the tax is the notion that the administrative costs of levying it are excessive and thus unsustainable. According to one lawmaker, the costs of levying it make up half of the revenue it generates (Wolfgang Schäuble 1996–04-26).

Social Democrats follow a very different set of arguments to make their case against abolishing the wealth tax in this early phase. They argue that this will privilege the rich and hurt the weak. To do so, they draw on a specific image of society as divided into those at the top and those at the bottom. One social group invoked to achieve this visual logic of contrasting, is, somewhat surprisingly perhaps, the family. The term “families” is among the most significant terms in this corpus relative to conservative discourse (see Fig. 2). Social Democrats use it to argue that conservatives privilege the rich on the back of families (thus also undermining their own values)^23^ by proposing to lower taxes on capital and at the same time, refusing to expand child allowances in a time of economic distress and high unemployment, especially in Eastern Germany. Fiscal policy must have a “stronger family component” (Klaus Hagemann 1997–11-25). More generally, the juxtaposition of the “top” and the “bottom” of society is central to the social justice frame: One MP calls the proposed tax reform a “crusade” into the wallets of “ordinary” people (Ingrid Matthäus Meier 1996–11-07). While this rhetoric achieves to invoke a contrast between the *deserving* bottom and the *undeserving* rich, it does not specify the role and the functioning of the wealth tax alleviating this situation. In this way, the social justice frame, if evocative, remains rather abstract.^24^

Another line of attack advanced by Social Democrats is that the abolishment of the wealth tax reduces state revenue, which hurts federal states’ budgets, in particular. After 1 January 1997, when the wealth tax was formally suspended, the debate shifts towards its possible reintroduction. The parliamentary roles also shift during this time, as Social Democrats move out of the opposition and assume the role of leading a coalition with the Green Party in 1998. Led by chancellor Gerhard Schröder, SPD does not draft a proposal to reinstate the tax. After the newly minced finance minister Oskar Lafontaine’s resignation in March 1999, the SPD adapts an increasingly conservative fiscal policy (Mudge, 2018: 359; Zohlnhöfer, 2004: 109–111). The main argumentative patterns on the wealth tax found in the parliament debates in this phase by and large remain the same; however, the debate subsides markedly after 1997.

### Phase 2, 2002–2007: Muted Exchanges

The second phase spans from the early to the mid-2000s, a time during which the governing coalition of Social Democrats and Greens continues to implement “third way” reform packages (including major reforms of income taxation, pensions, and unemployment provision). By 2005, a coalition of Conservatives and Social Democrats led by chancellor Angela Merkel (CDU) is in power. The same year, the newly formed alternative socialist party Die Linke, a fusion of the former Socialist Party of the communist-ruled German Democratic Republic and a branch of disappointed former SPD members (see note 5), enters parliament, bringing a new voice in favour of a wealth tax to the table.

From 2005 to 2009, CDU/CSU and SPD form the governing coalition. Phase 2 is marked by a muted conversation about this topic. In fact, here, the term “wealth tax” predominantly carries a negative connotation among both Conservatives and Social Democrats. They seem to agree that it is an unfeasible instrument. One important dynamic in this regard is arguably Social Democrats’ desire to demarcate their own position from that of Die Linke, which they deem as too radical.

In contrast to Phase 1, in this period, the wealth tax is not so much discussed substantively as it is cited as an example for a somewhat irrational fiscal policy.^25^ The debate is, in fact, again triggered by a conservative attempt to further reduce to role of capital taxes: In 2002, CDU/CSU propose to eliminate the wealth tax from the constitution altogether, but the effort remains unsuccessful. To conservative MP Michael Glos, raising the prospect of a wealth tax is part of a broader irrational agenda to “expulse capital” from Germany (“Kapitalvertreibungsprogramm”, 2003–11-26).

**Fig. 3:**
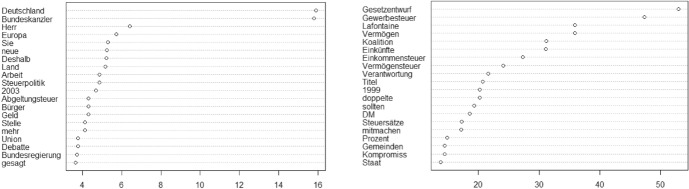
Chi-square results phase 2 (left: CDU/CSU, right: SPD)

Conservatives indulge the fact that Social Democrats are undecided about whether they want to reintroduce the tax or not. They repeatedly remind chancellor Gerhard Schröder (“Bundeskanzler”, see Fig. 3) that he has argued against doing so himself. MP Michael Meister mocks the “babble of voices” (“Stimmengewirr”, 2002–12-19) among Social Democrats on this topic, stating in one debate in 2003:

We just a learned it a few minutes ago: The federal parliamentary group of the SPD wants—unlike the chancellor, who proclaims the opposite in public—to introduce the wealth tax in Germany (…) I take note: There is a dissent between the chancellor and the parliamentary group that purportedly supports him. Every seven minutes, a business vanishes in Germany! (Michael Meister, 2003–04-03).

Social Democrats are wavering. They reject the conservative proposition to change the constitution and some segments of the party flaunt the possibility of reintroducing a wealth tax (“Gesetzentwurf”). However, party leader and Chancellor Gerhard Schröder avoids the issue altogether. He never mentions the wealth tax in the federal parliament. Overall, unlike in Phase 1, Social Democrats do not activate a social justice frame in favour of the tax. In the course of the debate on the CDU/CSU’s proposal to eliminate the wealth tax from the constitution, some social democratic MPs express bitterness about how Conservatives used the constitutional court ruling for their political goals to abandon the tax in the late 1990s. In 2003, one young social democratic MP from Bavaria, Florian Pronold, uses this opportunity to make a case for reinstating the tax (invoking the associated term “wealth”, see Fig. 3). Pronold explains that such a tax affects only asset holders at the top end of the distribution and that the decision to abolish the tax systematically hurt middle-class home owners, who were confronted with dramatically rising property taxes afterwards. He asserts that the actual costs of levying such a tax only amount to about a tenth, instead of a third, of its revenue (Florian Pronold, 2003–04-03).

This speech, however, is arguably an outlier in the social democratic camp during this phase. In 2005, after the alternative socialist party Die Linke puts forward a proposal for taxing wealth at a 5% rate, Social Democrats are keen on distancing themselves from this plan. In April 2006, for instance, social democratic MP Peter Friedrich who hails from a wealthy southern part of Germany spells out his opposition to it:

Whoever keeps on emphasizing in this debate that we must finally seize the rich by the collar to finance that—this is the language you are using—, and, at the same time, puts proposals about a wealth tax that envisions taxing from 300.000 Euro upwards on the table, should go and explain to a railwayman’s widow, who, for instance, has built her modest house together with her husband with her own hands in Radolfzell—my constituency—what this might mean to her! (Peter Friedrich, 2006–04-06).

Here, Friedrich invokes the conservative frame that the wealth tax hurts morally *deserved* wealth. SPD also draws on the globalization and tax competition argument to draw a boundary to Die Linke. In 2007, social democratic MP Rainer Wend reminds those on the left that we live in a “world of (…) tiny corporate taxes” where firms are “lured to Ireland (…) and Slovakia” and that one therefore needs to think twice before flaunting these kinds of ideas (Rainer Wend, 2007–02-01). In sum, then, the combination of contradictory positions on this issue and a lack of substantive arguments about the possible goals and applications of such a tax makes the social democratic position appear particularly weak during this phase. The muting of this issue is also reflected in popular opinion at the time. In 2007, according to one survey institute, only 35% of the German population holds a favourable view of reinstating the tax (Empter & Vehrkamp, 2007: 12).

### Phase 3, 2008–2016: An Uneasy Resurrection

The period after 2008 is marked by efforts to grapple with the ramifications of the global financial crisis and as well as the Eurocrisis. From 2009–2013, Germany is ruled by a coalition of Conservatives and Liberals, after which a coalition of Conservatives and Social Democrats takes power. With the return of the Social Democrats to power, renewed efforts to push for a wealth tax again subside.^26^

**Fig. 4:**
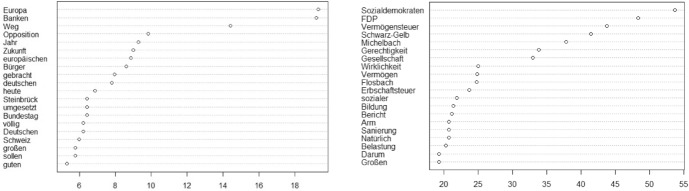
Chi-square results phase 3 (left: CDU/CSU, right: SPD)

Given the newly intensified public debate about economic inequality in Germany in the wake of the financial crisis, Conservatives find themselves pressured to defend their rejection of capital taxes with renewed vigour. One strategy to do so is to return to the argument that capital is the lifeblood of growth in the economy and to refine this view as part of a German approach to the European challenge to cope with the ramifications of the economic shock. The crucial reference to “Europe” (see Fig. 4) distinct for conservative speech in this phase portrays the German path as the “growth engine” (“Wachstumslokomotive”, Gerda Hasselfeldt, 2012–10-18) of Europe. “European” is also the most frequently used adjective in the CDU/CSU corpus of this period. According to conservative MP Olav Gutting (2012–11–28), the German path is a model for all of Europe, as tax revenues are “gushing” from plentiful sources—Gutting invokes the image of a rejuvenated society after the crisis, a situation in which an obstruction to the flow of capital such as a tax on wealth would “hit our mid-tier, low-yield firms (…) particularly hard”. Conservatives repeatedly define the “path” (see Fig. 4) out of the crisis in this way as the path of stability, a notion that is also associated with tight public debt discipline.

In another sense, the reference to Europe also serves as a warning. In dramatic and sinister language, MP Matthias Middelberg argues that the cases of France and Spain prove that taxes on capital cause unemployment to rise:

If there happen to be two, three bad years, firms will have to pay this from their capital base. The ones who are going to bleed, then, are the employees, because the jobs will be gone. We have to make sure this won’t happen. It would be a catastrophe. I can only tell you: You are on an entirely wrong track. In Europe, the French and the Spanish are the only ones who have a wealth tax. They also have higher tax rates, precisely what you are also aiming for. Thanks to this way of doing politics, the French today have an unemployment rate twice as high, and youth unemployment is three times as high. (2013–04-26).

Thus, in this period, CDU/CSU returns to its earlier economic frame that also invokes bodily metaphors, again charged with an acute sense of collective threat. The need to keep a wealth tax at bay is linked to a semantic field of vulnerability, fragility, and the need for protection, for an organic unity of “closed ranks” between employees and employers on this question (“Schulterschluss”, Peter Aumer, 2011–02-25). While conservative MPs’ attacks are often directed at the alternative socialist Die Linke, they deliver the same political message to the Social Democrats, their coalition partner after 2013.

CDU/CSU also sends contradictory messages with regard to how, exactly, the wealth tax relates to the principle of merit and to the legitimate sources of capital accumulation. On the one hand, conservative MPs argue that a wealth tax means “an encroachment into that which people have built up in a life’s worth of tiresome labor (…) your redistributive ideology destroys the willingness to be productive” (Hans-Peter Friedrich, 2010–09-15). On the other hand, however, the legitimate relation between productivity and capital growth seems less clear for the case of large firms. Conservative MP Christian von Stetten, who refers to the wealth tax as “poison” to society, argues that it would lead to.

Distortions of competition that would benefit listed companies and disadvantage family businesses. The large DAX-listed companies wouldn’t have trouble with the introduction of wealth tax, and a doubling of the estate tax wouldn’t bother the DAX-listed companies, either. But our mid-tier firms, the family businesses, they will have to include those additional costs into their budget. (Christian von Stetten 2013–04-25).

He insinuates that firms that fall outside of the category of “our mid-tier” “family business” will disregard the rules anyway, as a wealth tax would not even “bother” them.^27^ This, however, contradicts the idea of growth as a natural process as much as it departs from the notion that there is a balanced relationship between productivity and economic success.

Social Democrats, in turn, seek to carve out a distinct position on the issue for themselves (see Fig. 4). This is, again, born from the need to delineate a clear position vis-à-vis the leftist Die Linke, whose proposal to enact a 5% tax on wealth still haunts parliamentary debates in this phase, which strikes social democratic MP Nicole Kressl as “absurd” (2010–01-29) because, as she claims, a tax on wealth cannot be all about “envy, but about a fair distribution of duties and opportunities in our society.”

Social Democrats also return to a vivid, image-rich illustration of why they want the tax–this time, they are not afraid to identify with the label, and root the need for a wealth tax in social justice concerns. In fact, the term “justice” now appears among the top distinct words in the neighbourhood of the wealth tax (see Fig. 4). Social Democrats argue that the distribution of assets in society is skewed that the “balance” in society has tilted. MP Manfred Zöllmer invokes the image of a “widening social gap”^28^:

We need a wealth tax in Germany because a lot of people (…) derive their income not just from labor, but also from assets; the social gap is widening ever more. It’s a command of social justice. Our tax revenue must be distributed in a more just way. For that it is necessary to have a wealth tax, because the income and wealth distribution in Germany is deeply unjust. The upper 10 percent own more than 60 percent of all wealth in Germany. (Manfred Zöllmer, 2012–03-23).

To affirm the notion that a wealth tax is a matter of social justice, Social Democrats also make clear that they are, unlike the Green party, not in favour a one-time levy on wealth, but instead call for a regular tax, since only the latter can be an “adequate reaction to the structural problems” of the “growing gap between rich and poor” in society (Lothar Binding, 2012–09-27). Social Democrats, in this phase, also seek to appropriate the language of growth by asserting that “justice and growth are closely interconnected (Carsten Sieling, 2011–03-25). A wealth tax fuels growth, according to Sieling, by securing better wages and stimulating demand. In making such claims, however, Social Democrats are careful to emphasize that their proposal will “not hurt business assets” (“Betriebsvermögen schonen”, Lothar Binding, 2012–09-27), thus using the conservative language of an organic threat posed by the wealth tax. Surprisingly perhaps, Social Democrats do not reference the financial crisis or the Eurocrisis as an immediate cause for reinstating the tax.

An important co-occurring theme in this phase is the notion that a wealth tax will have a critical role in providing the financial means to foster public spending on education (Nicolette Kressl 2010–01-29). The argument is that redistribution is necessary to finance social investment spending. This is linked to arguments about how welfare policies must aim at creating a levelled playing field, and work towards equality of opportunity, notions that are typical of the transformed social democratic profile of the 2000s on a European level (Gingrich & Häusermann, 2015).

In sum, then, until 2013—when the SPD returns to power, again in a coalition with CDU/CSU—, Social Democrats are committed to framing this issue as future-oriented, inclusive, and reasonable.^29^ CDU/CSU, on the other hand, return to their earlier, gloomy narrative about how the path of growth and stability is endangered by such considerations, and how the health of the economy as a whole is at stake in this debate. It is, then, an uneasy resurrection of this topic, as the conservative frame seems to predominate. Once Social Democrats enter government in 2013, the issue is quickly abandoned.

### Contesting Frames of Meaning

From an analysis of the relevant segments over time, it is possible to distinguish two overarching, dominant frames of meaning that are variously activated over the course of the period 1996–2016 in parliamentary debates on the wealth tax: First, an efficiency frame that is centrally structured around growth (concerned with a mechanism of growth and rooted in a neoliberal idea of “market justice”, Lane 1986), and second, a social justice frame that is concerned with redistribution. Key elements found in those frames are summarized in Table 3.

**Table 3:** Two dominant frames

	Efficiency/growth frame	Social justice/redistribution frame
Wealth tax	Shrinks investments, diminishes competition Hurts capital and thus labour—a negative “trickle down” effect Illegitimately targets “deserved” wealth Is too expensive to administrate	Strengthens the position of weak groups in society (families, low-income individuals) Can be a basis of social investment spending, especially education. Growth and redistribution are not mutually exclusive Can be moderately implemented
Object	Economy as a whole	Particular groups in society who are “drifting apart”

While Conservatives stick to the efficiency frame over the entire time period, Social Democrats’ arguments are not limited to the social justice frame. In some cases, they borrow from their political opponent’s semantic repertoire. In other cases, they are not internally consistent in the way they apply the social justice frame. What is more, Social Democrats rarely provide details about *who* will be affected by such a tax: They very rarely come close to describing the mechanisms of capital accumulation in place at the top end of the wealth distribution. This allows Conservatives to portray mid-tier companies and family businesses as the backbone of the German economy and to argue that the wealth tax inflicts damage on the economy as a whole.

According to Goffman (1974), frames point to a larger context of meaning. In our case, then, one overarching problem that is at stake in the debates about the wealth tax in German parliament during this period is really a fundamental exchange about how economic value is created in society. Conservatives and Social Democrats construe this object differently. While Conservatives reference the wealth tax to talk about the economy as a whole—pointing squarely at a comprehensive mechanism of growth in it—, Social Democrats reference it to illustrate that particular groups in society are “drifting apart”. Particularly compelling, perhaps, is the evocative language introduced by Conservatives in this regard: In conservative speech on the wealth tax, the economy is like an organism that must be protected from harmful interventions. Capital is the principle of vitality, it is what keeps this organism alive. This is why, as conservative MPs like to point out, capital taxes are “poison” to the economy—in the sense of an alien substance that causes great harm, not just to single organs, but to the system as a whole. The efficiency frame construed around the wealth tax, then, is firmly rooted in a biological imagery. Therein, it resembles the kind of “social naturalist” imagery that Somers and Block (2005) reconstruct in conservative welfare critics’ positioning in US-American welfare reform debates in the 1990s.

## Discussion and Conclusion

In this article, we traced how the two major political parties in Germany, the conservative Christian Democratic Party (CDU) and the Social Democratic Party (SDP), debated the wealth tax in the German federal parliament between 1996 and 2016. This debate was initiated by the abolishment of the wealth tax following a ruling by Germany’s highest court that declared the unequal treatment of real estate and financial wealth unconstitutional. While the wealth tax has been subject to controversial discussions thereafter, no serious political effort was made to reintroduce it.

Our analysis shows that both the Christian Democrats’ and Social Democrats’ positioning towards the wealth tax largely follows these parties’ established policy positions on egalitarian public policies more generally (Banting, 1991; Esping-Andersen, 1990; Korpi, 1983). While the CDU framed its opposition against the taxation of wealth from an efficiency perspective, the SPD variously attempted to mobilize a social justice frame in order to generate support. Similar to the findings on media debates presented by Lichtenstein et al. (2016), we find that these two frames exist amidst a set of more technical considerations around the existence of such a tax and that the antagonism between them really seems to constitute a substratum of this debate over time. However, our findings do not merely reflect straightforward partisan divides between the political representatives of capital and labour. More interestingly, especially Conservatives’ framing employs a strong evocative language and political symbolism. While the efficiency frame foregrounds the creation of jobs and the maintenance of competitiveness, it frequently draws on biological metaphors. In calling the tax “poison” to the economy, Conservatives narrate the wealth tax as a threat to the social body as a whole, arguing that society has to be protected from its detrimental, sickening effects. Social Democrats, in turn, only selectively achieve to activate the social justice frame. They rarely account for why taxing wealth would be in the interest of society as a whole, instead of particular groups in society. Perhaps paradoxically then, for the time period investigated here, conservative discourse succeeds in linking opposition to the wealth tax to a principle of social cohesion (qua economic integration). Thus, one important lesson from our analysis is that the very meaning of the wealth tax can be construed in various ways and to various ends (cf. Prabhakar, 2008). The outcome of this discourse, is, to be sure, highly consequential: In the period observed here, Germany has seen an ever-increasing concentration of wealth. It has further transformed into an export-oriented economy that channels the profits towards top incomes and asset holders whose savings rate is skyrocketing, while wages and investments have stagnated (Klein & Pettis, 2020).^30^

This resonates with recent insights offered by Rieder and Theine (2019) into the powerful role of metaphors in delegitimizing the taxation of wealth in newspaper articles. Surveying counter-arguments against policy proposals by French economist Thomas Piketty, the authors document an “array of metaphors [that] are used to mark the immediate danger that springs from them: Taxes are a ‘burden’, ‘cause pain’, ‘endanger life’, are a ‘curse’ and are often placed in military and war-like semantic contexts” (Rieder & Theine, 2019: 256). Evidently, these discursive resources operate across the institutional venues of parliament and the media.

Our analysis has focused on the voice of two major parliamentary groups on the left and on the right. Our method comes with the assumption that the debate about the wealth tax is structured in a way that resembles a semantic gap between political antagonists who each draw on a particular language to advance their cause. While the juxtaposition reveals powerful patterns in a longitudinal analysis, this focus also comes with limitations that future research must address: First, we need to know more about how changing party landscapes (and the rise of alternative leftist parties or the Greens, as well as the authoritarian populist right) affect this debate. Second, the frames and ideational alignments that define the meaning and the purpose of the wealth tax must be further studied in what Stephanie Mudge calls the “cultural infrastructure” of political parties (2018), that is, the network of interest groups, think tanks, economic advisors, and scientific experts that shape power alliances within parties.

Today, the debate on the wealth tax is returning in full steam. In 2019, the German Social Democrats have elaborated a new proposal for such a tax (SPD, 2019), and in 2020, the dramatic fallout from the coronavirus crisis has sparked a debate about how the massive public debt burden that results from the state’s historic relief effort can be fairly distributed in society. At the same time, the pandemic has further aggravated wealth inequalities. Given that times of crisis have historically often been occasions on which societies succeeded in taxing the rich more extensively (Scheve & Stasavage, 2016), what are the implications of our analysis for the present situation? If our interpretation of the conservative framing of the wealth tax as a threat to societal vitality has some plausibility, attempts at reinstating a personal net wealth tax would need to point out why this would serve not only the less-privileged groups in society, or the man on the street, but rather society as a whole. The Covid-19 pandemic, and the resulting fiscal and economic burden it entails, is a moment of contingency: It could be apprehended as an instance of an external shock that seriously endangers the social fabric as a whole, which would then require the mobilization and contribution of all members of society. But other outcomes are also possible. The narratives about this crisis and its ramifications that emerge as dominant will likely also shape fiscal policy for years to come.

## Appendix

**Table:**

	Parliamentary fraction	Total Number of speakers in this legislative period	Number of speakers debating the wealth tax (according to our criteria)	Percent (%)
Legislative period 13, 1994–1998	CDU/CSU	263	14	5
	SPD	245	16	7
Legislative period 14, 1998–2002	CDU/CSU	248	3	1
	SPD	298	2	1
Legislative period 15, 2002–2005	CDU/CSU	251	12	5
	SPD	239	2	1
Legislative period 16, 2005–2009	CDU/CSU	224	0	0
	SPD	214	6	4
Legislative period 17, 2009–2013	CDU/CSU	231	14	6
	SPD	153	9	6
Legislative period 18, 2013–2017	CDU/CSU	302	0	0
	SPD	198	1	1

## Footnotes

1 While wealth can also be taxed based on its increases (e.g., capital gains) or its transfer (e.g., inheritance or inter vivo transfer), we focus on the regular, recurring taxation of the stock wealth in the form of an annual, personal net wealth tax (Schratzenstaller, 2011). Here, all of a person’s wealth—including financial wealth, equity interests (shares or property rights), as well as property (real estate, property, and valuables)—is added, and liabilities and debts are deducted (Schratzenstaller 2011: 7).

2 Historically, wealth taxes are among the oldest taxes in the world. In an early form, taxes on property have already been collected during antiquity (Wieland 2003). With the emergence of modern nation-states and the strengthening of their administrative capacities, income taxes have come to replace wealth taxes as the primary source of fiscal revenue (Seelkopf et al., 2019).

3 Tax rates of an annual personal wealth tax are usually set in such a way that tax payments can in principle be settled by current investment income. Thus, unlike with inheritance taxes or one-time property taxes—and despite what is sometimes assumed in the public debate—, the aim of a personal net wealth tax is not to reduce the stock of wealth.

4 Frames “‘define problems, diagnose causes’, ‘make moral judgements’ ‘suggest remedies’” (De Vreese and Kandyla, 2009, quoted in Closa and Maatsch 2014: 830). They contain elements of narrative structure (Somers, 1994) such as evaluation or causal attribution. Frames can also be enriched with symbolic and metaphorical language.

5 The Left Party resulted from a fusion of the successor of the Socialist Party of the German Democratic Republic and a group that separated from the Social Democrats due to their opposition to a series of welfare reforms introduced by the Red-Green administration under Chancellor Gerhard Schröder.

6 Within this context, the reintroduction of the personal net wealth tax—which had since 1997 been demanded by the Social Democrats, the Green Party, and the predecessor of the left party—was not followed up on (Zohlnhöfer, 2004: 110). As we are not aware of prior studies that analyse the—possibly shifting—positioning of the SPD towards the wealth tax, our analysis also contributes to the study of patterns of ideational realignment within left parties during the late 1990s and early 2000s (Giddens, 1998; Gingrich and Häusermann, 2015; Mudge, 2018; Zohlnhöfer, 2004).

7 GermaParl, which comprises more than 100 million tokens, is a structurally annotated corpus: Meta-data like name of speaker, party and parliamentary group affiliation, and date of speech, are included in the corpus and can be analyzed using an R package developed for this corpus (“PolmineR”). See www.polmine.de

8 To narrow down the analysis, we focus on periods (observed at the level of single dates and speakers) during which the term “wealth tax” appeared more frequently. We spell out the criteria when introducing the sub-corpus further below.

9 Hence, we are not interested merely in frequencies—numbers of times the word is being mentioned around others—, but also in patterns of relational associations. We follow the basic structuralist assumption that a word derives its meaning in relation to others. Different linguistic functions may be associated with neighboring words.

10 Computational social science is a rapidly expanding field. It is marked by the use of large datasets (which would exceed a single researcher’s capacity to code manually) and by the application of mathematical or algorithmic models to trace and classify meanings in them. In text analysis, computational social science tools allow to “discover novel patterns in text data on the basis of similarity, structural association, or predictive power. These patterns may surprise the social analyst and merit interpretive scrutiny and labeling, provoking new social theory to explain them” (Evans and Aceves, 2016: 25). We make use of the pre-existing functions included in the GermaParl/PolmineR package in R (Blätte and Blessing, 2018), which is itself programmed as a text-as-data approach. Hence our results are transparent and easily replicable.

11 It ranks behind eco-tax (“Ökosteuer”, 4130), but appears a similar rate as VAT (“Mehrwertsteuer”, 1898), business tax (“Gewerbesteuer”, 1795), and more often than income tax (“Einkommensteuer”, 1255) and corporate tax (“Körperschaftsteuer”, 660). There are some variations of the main word that are, however, not included in the statistical analysis, such as the phrase “levy on wealth” (“Vermögensabgabe”, 308) and the phrase “wealth-taxation” (“Vermögensbesteuerung”, 103). Phrases like “taxing wealth (“Besteuerung von Vermögen”, 17), “taxes on wealth” (“Steuern auf Vermögen “, 4) are rarely used and excluded them from the analysis. Adjective constructions involving the wealth tax are very rare. The related phrase “millionaire tax” (“Millionärsteuer”, 65) is excluded as well.

12 Smith Ochoa and Hugendubel (2019), Theine and Grisold (2020) find similar trends analyzing newspaper debates on economic inequality and the wealth tax.

13 To understand how the choice to delineate three phases affects our results, we also computed the statistical analysis for the six legislative periods (LP) between 1998 and 2016 and compared the two approaches. The results are very similar: LP 13 (1994–1998, although only 1996–1998 are captured in the GermaParl data) corresponds to phase 1, LP 15 (2002–2005) corresponds to phase 2, LP 17 (2009–2013) corresponds to phase 3. LP 14 (1998–2002), LP 16 (2005–2009), and LP 18 (2013–2017, with 2016 as the final year in this dataset) each generate only a very small corpus, and we find little to none distinct language associated with the term “wealth tax” in them.

14 Our analysis dos not focus on individual Members of Parliament, but it is possible to understand this evolution at the level of speakers. It turns out that, for both parliamentary groups, between 0 and 7% of all the speakers in a given legislative period take up this topic according to our criteria (see below). Social Democrats do not feature a broader range of speakers on the topic than Christian Democrats. We summarize these results in the appendix.

15 Blätte and Wüst (2017) use the same measure to analyze how legislators whose parents are not native German citizens use a different language than their peers when talking about migration. The Chi-Square test is consistent with the way the available metadata is structured in the PolmineR package and can be performed using its pre-existing “frequency” function (Blätte and Blessing, 2018).

16 In corpus linguistics, a “token” is the smallest linguistic unit in a given corpus; in our case, tokens are words. We applied a Chi-Square test to compare each of the six corpora to the entire corpus of the respective phase. In each, the term “wealth tax” scored the highest Chi-Squares values, so we treat the subcorpora as defined by this topic on these grounds.

17 To identify relevant segments, we apply a keywords in context (KWIC) search of a word gained in this way, defining a maximum distance of 100 tokens from this word to the term “wealth tax”.

18 The words identified by the Chi-Square test inform the qualitative analysis in two regards: They often (though not always) carry distinct meanings in themselves, and they index particularly relevant segments. A more conventional approach would lead us to analyze entire speeches and then interpret and synthesize the meaning gained from these larger entities. In our approach, by analyzing smaller segments of speech identified via the keywords, we focus on the meanings that are conveyed in ways of speaking that are characteristic for each of the two parties. Hence, we foreground language that is actively claimed by the actors.

19 Relatedly, we find references to social processes that would either be *amplified* or *weakened* if the wealth tax was in place.

20 This is also a particular form of speech: A reminder about how the world works, a wake-up call in the name of reason: Capital is mobile in a world of global competition—and proponents of the wealth tax seem to *ignore* this reality. Already, businesses are “fleeing” Germany, as one conservative lawmaker puts it.

21 The term “jobs” also appears significantly more often in the CDU/CSU corpus phase 1 than in all speeches by all parliamentary groups in this phase.

22 The logic of this semantic field is apparent also in the adjectives used by Conservatives in this phase: Words like “investments”/ “invested” and “mid-tier” are distinct to CDU/CSU speech in this period.

23 MP Lydia Westrich attacks Conservatives by stating that chancellor Helmut Kohl (CDU) had for years labelled the family as the “seedbed of the state” and yet failed “to provide that seedbed with adequate nutrients” and thus has “lost the trust” of families (1999–01-22).

24 There are two exceptions, both of which are however mentioned in passing and can thus not be regarded as essential to Social Democrats’ arguments at the time. This is, first, a problematizing of the specific mechanism of capital accumulation (the rate of return on capital as an unjust advantage for those who are already in possession of it) noted briefly in speeches by Ingrid Matthäus Meier (1996–06-14) and Erika Lotz (1997–03-13), and second, introducing evidence to problematize and reject the key conservative claim that that capital, in the form of investments in and within firms, will trickle down to generate and sustain jobs and ultimately higher wages (Detlev von Larcher 1996–09-19). The economic problems in the former communist East of Germany—where the wealth tax was never levied during the 1990s—serves as a case in point that an absence of such as tax does not attract investments.

25 Note the low Chi-Square values for CDU/CSU in Fig. 3. From an analysis of legislative periods 3 and 4 (2002 to 2005 and 2005 to 2009), we gain a similar picture: There is not much distinct language associated with the term in this period, even if more speakers by CDU/CSU are active in legislative period 15 and more speakers by SPD are active in legislative period 16 (see table in the appendix).

26 In this phase, the Green party introduces a proposal for a one-time levy on wealth.

27 Saez and Zucman (2019) identify this as an important, if deeply cynical, trope among opponents of capital taxes: The idea that companies will find ways to avoid them no matter what, which effectively suggests that political action is futile.

28 The German “soziale Schere”, the image of a widening scissors, which has somewhat of a violent connotation in German.

29 Unlike Conservatives, they invoke positive adjectives—like “social”, “reasonable”, “important”—in their speech (results for adjectives are not shown).

30 In line with this observation, we suggest that future research will find it fruitful to analyze discourses about the wealth tax as part of a semantic ecology of related taxes, such as the estate, the gift, and the inheritance tax.

## Declarations

### Data Availability

The data that support the findings of this study are openly available in the GermaParl R package and the GermaParl CWB corpus at Blaette, Andreas (2020): GermaParl. Download and Augment the Corpus of Plenary Protocols of the German Bundestag. R package version 1.4.1. https://CRAN.R-project.org/package=GermaParl and Blaette, Andreas (2020): GermaParl. Linguistically Annotated and Indexed Corpus of Plenary Protocols of the German Bundestag. CWB corpus version 1.0.6. https://doi.org/10.5281/zenodo.3735141

### Code Availability

Code for the analysis is available from the authors upon request.
